# First Exploratory Study on the Ageing of Rammed Earth Material

**DOI:** 10.3390/ma8010001

**Published:** 2014-12-23

**Authors:** Quoc-Bao Bui, Jean-Claude Morel

**Affiliations:** 1 University of Savoie, Laboratoire Optimisation de la Conception et Ingénierie de l’Environnement, CNRS, Polytech Annecy-Chambery, 73000 Chambery, France; 2 University of Lyon, LTDS, CNRS, LGCB, Ecole Nationale des Travaux Publics de l’Etat, 69120 Vaulx-en-Velin, France; E-Mail: jean-claude.morel@entpe.fr

**Keywords:** sustainable development, rammed earth, ageing, *in-situ* measurement

## Abstract

Rammed earth (RE) is attracting renewed interest throughout the world thanks to its “green” characteristics in the context of sustainable building. In this study, the ageing effects on RE material are studied on the walls which have been constructed and exposed for 22 years to natural weathering. First, mechanical characteristics of the “old” walls were determined by two approaches: *in-situ* dynamic measurements on the walls; laboratory tests on specimens which had been cut from the walls. Then, the walls’ soil was recycled and reused for manufacturing of new specimens which represented the initial state. Comparison between the compressive strength, the Young modulus of the walls after 22 years on site and that of the initial state enables to assess the ageing of the studied walls.

## 1. Introduction

Rammed earth is an ancient building material that is benefiting today from a renaissance due to its sustainability. The materials are sandy-clayey gravels soils which are compacted inside a formwork. The soil composition varies greatly but should not include any organic components. Compaction is done at the soil’s optimum water content that provides the highest dry density for the given compaction energy [1]. The rammed earth wall is composed of several layers. For each layer, the soil is poured about 15 cm thick into a formwork and then rammed with a rammer (manual or pneumatic). After compaction, the thickness of each layer is typically 8–10 cm. The procedure is repeated until completion of the wall. A detailed presentation of rammed earth construction can be found in Walker *et al.* [2].

For traditional rammed earth construction, referred to as “rammed earth” (RE) or “unstabilized rammed earth”, the only binder is clay. Other binders can also be added such as cement or lime. This is often called “stabilized rammed earth” (SRE). The main advantage of stabilization is to increase the durability and mechanical performance. However, stabilization increases the construction cost and environmental impact.

Rammed earth is the focus of recent scientific research for three main reasons. Firstly, the earthen construction is sustainable because it uses a natural and local material [3]. Secondly, the earth material can act as a natural moisture buffering of indoor environments [4]. Finally, the number of historic rammed earth buildings in Europe and in the world is still significant [5,6]. Maintaining this heritage needs scientific knowledge to apply appropriate renovations.

Several research investigations have recently been conducted to study the characteristics of rammed earth: durability and sensitivity to water [5,7,8]; compressive mechanical characteristics [1,9,10,11]; pullout strength [12]; shear strength [13,14,15]; dynamic behaviour [16,17]; capacity subject to lateral wind force [18,19]; thermal properties [20,21]; hygrothermal properties [4,22] and living comfort [23]. However, to our knowledge, there is not yet any scientific study on the aging of rammed earth. Indeed, the famous phenomenon relative to the aging of old rammed earth structures is the process of buckling [24].

**Figure 1:**
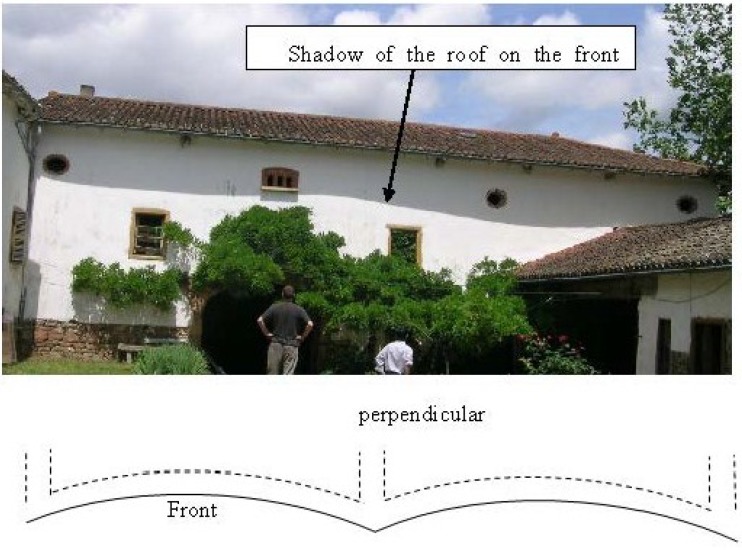
A rammed earth wall more than 200 years old. Upper: front view; bottom: plan.

Figure 1 presents an example of a rammed earth wall which is more than 200 years old. Note that the shadow of the roof on the front wall is not a horizontal straight line. This is due to the horizontal buckling of this wall that can be seen on the plan (Figure 1, bottom). The buckling phenomenon depends on several parameters (wall’s slenderness ratio, eccentricity of the loading, boundary conditions, …) but an important parameter which relates to material’s characteristic is the creep phenomenon.

Indeed, the critical Euler buckling load F_c_ is given by the formula:

F_c_ = π^2^ EI/l_0_^2^ (1)

where E is the Young modulus, I is the moment of inertia, l_0_ is the buckling length of the studied wall which was discussed in the Maniatidis and Walker study [11].

The moment due to the buckling phenomenon is a function of F_c_:

M = K∙M_0_ (2)

where K = F_c_/(F_c_ − F), F is the compressive load; M_0_ is the moment due to the initial deformation (M_0_ = F. y_0_, with y_0_: initial deformation).

When E decreases then F_c_ decreases, and M increases as well as the out-plane deflection of the wall.

It is usually observed on other geo-materials that the Young modulus varies following the time due to the ageing. Therefore, in this study, the ageing is also studied on rammed earth material. The studied rammed earth walls have been constructed and exposed for 22 years to natural weathering. Comparison of the compressive strength and the Young modulus between the walls after 22 years on site and that of the “initial” state specimens was carried out, that enables assessing the ageing of the studied walls.

## 2. Characterizing 22 Years Old RE Walls

### 2.1. Presentation of Studied Walls

**Figure 2:**
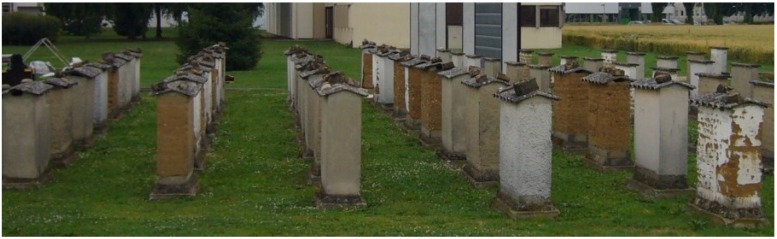
Rammed earth walls constructed and exposed for 22 years to natural weathering.

The wall specimens were built in 1985 (Figure 2) thanks to the Rexcoop program, controlled by the French Scientific and Technical Building Center (CSTB), near Grenoble, in a French Alpine valley, at an altitude of 212 m. The temperature of the site can vary from −20 °C to 38 °C for some particular years and its average varies from 2 °C to 20 °C. The annual rainfall is about 1000 mm, the direction of prevailing winds is NE-SW and the maximum wind speed is 21 m/s.

A total of 104 earthen wall specimens were built using rammed earth, straw-earth, compressed earth block (CEB) masonry, and vibrated-compressed block masonry. Different types of soils were used for each of these construction techniques. Different types of surface coating were tested. This paper focuses only on rammed earth walls. All rammed earth walls were protected by asbestos cement roof. More information on these walls can be found in Bui *et al.* 2009 [5].

**Figure 3:**
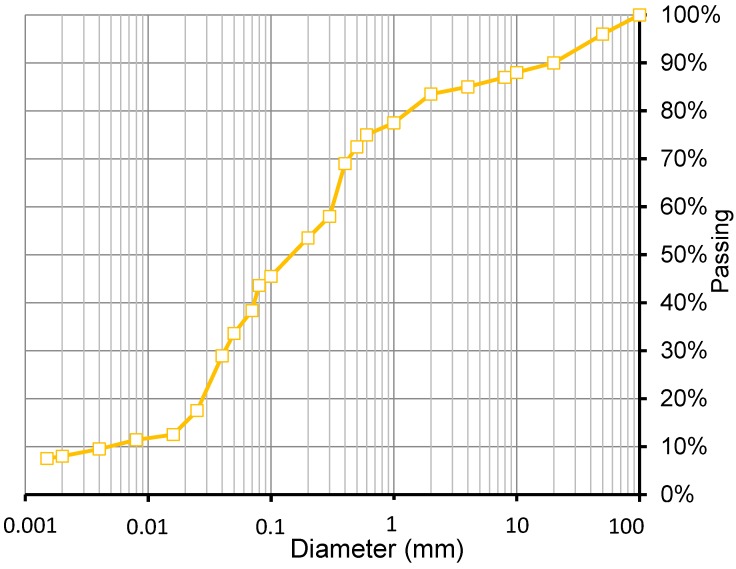
Grain size distribution of the used soil.

The rammed earth walls (1 m width × 1.1 m height × 0.4 m thickness) were manufactured on a concrete foundation with a 25 cm base exposed above ground level. A bituminous layer was painted on top of the base to prevent water from capillary rise penetrating into the RE walls. Local soil from a nearby site mixed with a cultivator was used for the RE walls. Three soils were used but in this study, only walls from one soil were investigated. Its grain size distribution is presented in Figure 3.

The manufacturing water content of the soil was about 10%. The metal formwork was assembled according to the wall dimensions (1 m × 0.4 m × 1.1 m high). Soil was poured into the metal formwork in 150 mm layers and then compacted with a pneumatic rammer. There was no control of the walls’ density.

The mechanical characteristics of these “old” walls were determined by two parallel approaches: firstly, *in-situ* dynamic measurements were carried out on the walls which enabled to identify the walls’ dynamic characteristics, and from that their Young modulus could be determined. Secondly, the studied walls had been cut, then these specimens were tested in laboratory to obtain the compressive strength and the Young modulus. Two approaches were carried out in parallel because the measurement of the Young modulus on the specimens cut from the walls was delicate [7]. The *in-situ* dynamic measurements could give a confirmation (or not) about the results obtained by the compression tests.

### 2.2. In-Situ Dynamic Measurement

#### 2.2.1. Measurement Device

**Figure 4:**
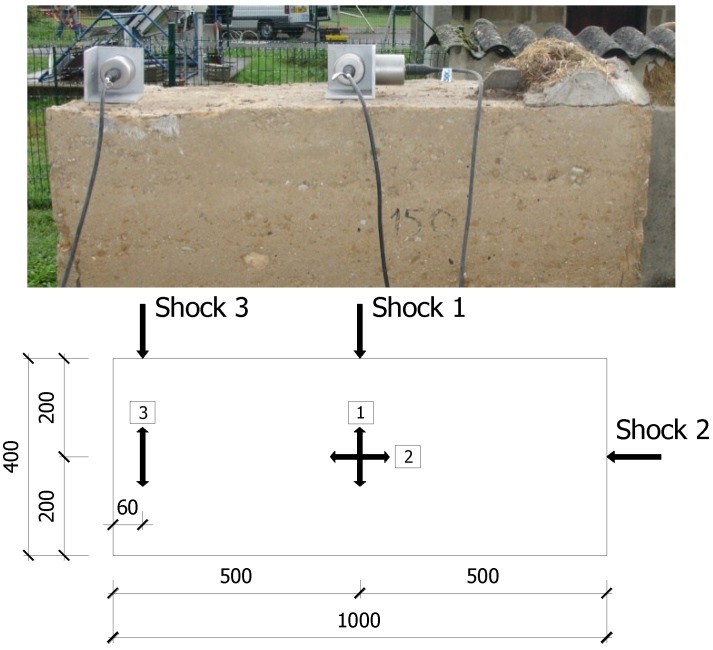
Arrangement of the sensors on the wall A (dimensions in mm).

Three accelerometer sensors with a sensitivity of 1 µg (with g being the gravity field equal to 9.8 m/s^2^) were placed on top of the wall (Figure 4): two sensors in the centre to measure two horizontal accelerations following the two main axes of the wall; and another sensor on the edge to measure possible torsional movements.

The excitation consisted in a light shock (by a hammer) which was applied to the top of the wall. Three configurations were carried out (Figure 4, bottom): (1) a center shock following the transversal direction of the wall; (2) a center shock following the longitudinal direction of the wall; (3) an offset shock following the transversal direction. These configurations excite the possible vibration modes of the wall: transversal, longitudinal and torsional.

#### 2.2.2. Frequencies Measured *in-Situ*

In the framework of this study, three RE walls were measured, 22 years after their construction. They had three different types of coating: the reference rammed earth—with no protection layer (wall A), the rammed earth protected with plaster (wall B) and the rammed earth protected with paint (wall C). Since the wall B was protected with plaster (thickness about 3–4 cm), the measurements were made after removing the plaster to eliminate its influence on the results. For the wall C protected with paint (thickness <0.5 mm), the measurements were performed without removing the protection, assuming that its contribution to the wall’s dynamic behaviour was negligible.

**Figure 5:**
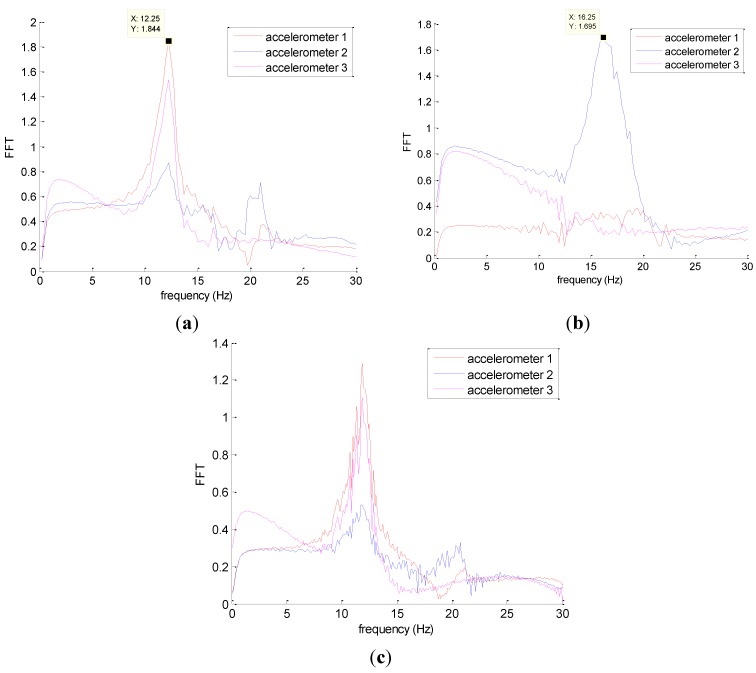
A result of wall A induced by shock 1 (**a**); shock 2 (**b**); and shock 3 (**c**).

Figure 5 shows typical results obtained after a signal processing for the shocks 1, 2 and 3. Each peak corresponds to a modal frequency. In this case, the frequency of the first transversal mode is identified at 12.25 Hz. For shocks 1 and 3, sensor 2 does not give a clear signal because in these cases, excitations were perpendicular to the sensor 2; there was no major vibration in the wall’s longitudinal direction. The result for sensor 3 is similar to that of sensor 1, since the torsion was not clearly captured (shocks were not important enough to solicit this mode). For shock 2, sensors 1 and 3 do not give significant information but sensor 2 captured the second vibration mode which is in the longitudinal direction. In this case, the second modal frequency is of 16.25 Hz. Results of two others walls are presented in Table 1.

**Table 1:** Frequencies measured and corresponding moduli identified by the model.

Walls	Moduli identified (MPa)	Measurements		Model	
		f_1_ (Hz)	f_2_ (Hz)	f_1_ (Hz)	f_2_ (Hz)
Wall A	104	12.25 ± 0.05	16.25 ± 0.12	12.25	16.48
Wall B	98	11.87 ± 0.08	16.20 ± 0.09	11.87	15.97
Wall C	90	11.38 ± 0.10	15.38 ± 0.22	11.38	15.31

#### 2.2.3. Finite Element Modelling (FEM) of the Walls

**Figure 6:**
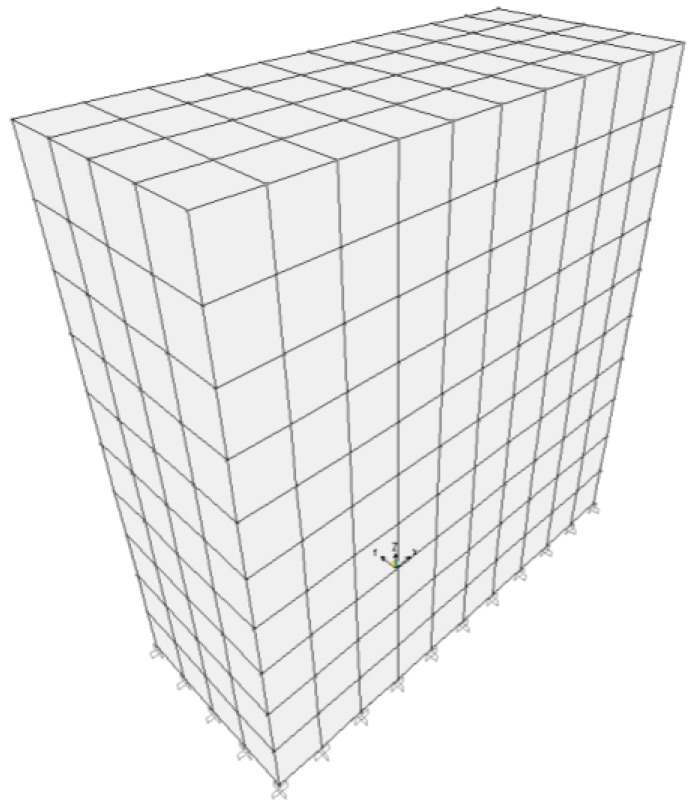
Wall A modelled with FEM.

The walls are modelled with solid elements (Figure 6). Following the dynamic of structures theory, for given dimensions, the natural frequencies depend only on the density and the elastic characteristics of the material. For the modelling, the material was assumed isotropic (which is acceptable for dynamic measurements which were performed in very small strain [1]). The Poisson’s ratio was taken of 0.22 following a previous study [7]. The nodes between the RE wall and the concrete foundation were restrained in translations. This boundary condition can be justified following the results presented in the previous studies (Bui *et al.* [1,16], Maniatidis and Walker [11]). The principle to determine modulus from natural frequencies was presented in Bui *et al.* 2009 [1]:

(3) $$frequency=function(dimensions,Poisson´s\;ratio)\sqrt{\frac{Young´s\;modulus}{density}}$$

**Figure 7:**
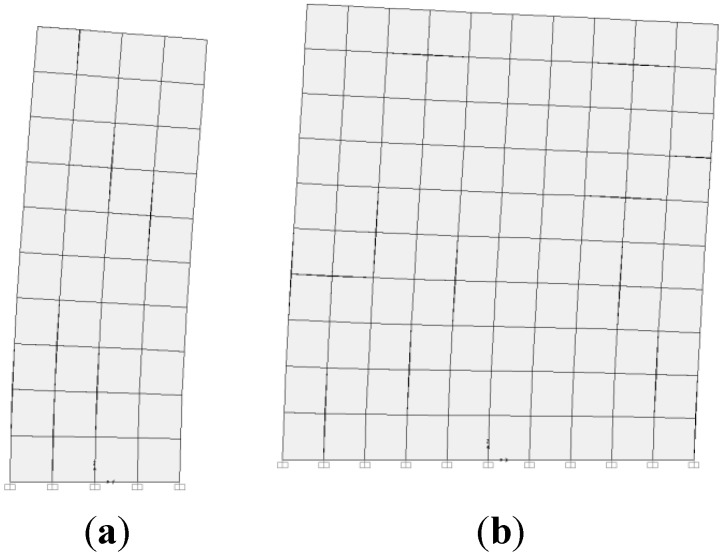
First vibration modes of the wall A. (**a**) First vibration mode, in the transversal direction; (**b**) second vibration mode, in the longitudinal direction.

The dry density was measured in laboratory and will be presented in Section 3.2. The results of the identified modulus are given in Table 1. The first main modes of vibration are shown in Figure 7.

From the results presented in Table 1, the Young moduli of the measured walls were about of 100 MPa. These values are lower than that indicated in the literature (for example, Bui *et al.* [1] had identified moduli about 450 MPa for unstabilised rammed earth). This value of the Young modulus will be compared in the next section with that obtained by compression tests.

**Figure 8:**
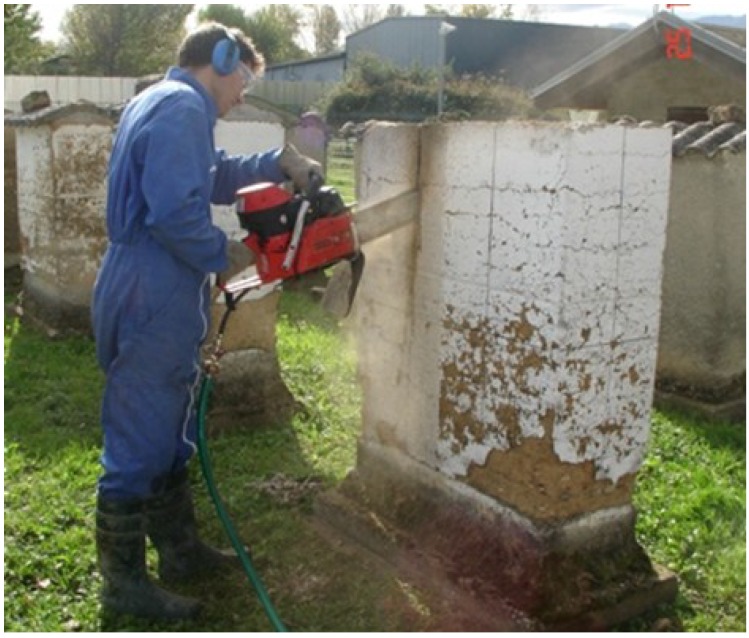
Cutting a wall with chainsaw.

Following these results, the walls’ modulus values are in the order: without protection layer (wall A) > protected by plaster (wall B) > protected by paint (wall C). The worst behavior of wall covered by paint can be explained by the following reasons. On one hand, following observations on site, this type of protection was thin and stuck well to the RE surface. With temperature changes, the paint layer and the RE deformed differently, but the cohesion between the paint layers and the RE surface was stronger than the internal cohesion of the paint which was very thin. Therefore, cracks appeared quickly in these paint layers and water could penetrate easily. On the other hand, the roof could only protect the wall top, the lower part was exposed to rain drops. Due to the cracks mentioned above, the lower part of these protections was washed away by rain (Figure 8). The disappearance of the protection layer at the bottom encouraged water penetration, moving upwards by capillarity. This water could not exit because the paint inhibited the open porosity of the RE. The RE became wetter (Hall and Djerbib [8]). Indeed, after 22 years, the surface quality of that wall is worse than that of the reference wall (wall A). However, due to the limited number of the walls in this study, this observation should be tested by other investigations on other walls.

## 3. Laboratory Static Tests

### 3.1. Cutting out the Specimens

The specimens were taken from the walls using a chainsaw generally used to cut concrete and stones (Figure 8). Its blade was 30 cm long, meaning that the total thickness of the wall (roughly 41 cm) had to be cut in two steps. The disadvantages of using this method to take specimens are the generation of vibrations and the use of water, which can decrease the mechanical strength of the specimens, notably due to the splitting apart of the layers of earth.

### 3.2. Density Measurement

Since the specimens taken from the walls were roughly shaped, their density was estimated using hydrostatic weighing, once they had been coated with paraffin to seal them. Three specimens approximately (8 × 10 × 20) cm^3^ in size—obtained from three different walls—were measured which gave a mean dry density of 1.82 ± 0.01. The dry density value of 1.82 is lower than that of the new RE presented in a previous study, by Bui *et al.* 2009 [1], which was around 1.92. This lower dry density will be discussed in the next sections.

### 3.3. Unconfined Compression Test

The specimens cut from the walls were transported to the laboratory and re-shaped with a table saw. Several specimens were cut from the walls, but only six specimens (obtained from the cutting of all walls) had an acceptable quality for the testing. Each specimen had dimensions of (16 × 16 × 27) cm^3^ with a slenderness ratio of 1.7. Three were tested in the direction perpendicular to the layers, and three others in the direction parallel to the layers. The specimens was dried naturally in normal atmospheric conditions before testing, so their moisture content was similar to that of the walls.

The method using extensometers to measure strains of the central part of the specimen which had been used in the previous study Bui *et al.* 2014 [7] was tried but it did not work here. The extensometer did not correctly detect the strains (no strain measured or important differences between three extensometers on a specimen). This could come from the prismatic form and the surface quality of the specimens which did not enable the extensometers to operate correctly. Therefore, the strain was calculated by dividing the vertical displacement of the press by the specimen height. Because this way did not prove to be the better method to measure the strain, the above dynamic measurements were performed to check the relevancy of the results obtained by the compression tests. On the other hand, the aim of this paper to compare the results obtained on the “old” and “new” specimens, so it is an ageing factor which is researched. Call E_h_: modulus measured on all height of the specimen; E_1/3_: modulus measured in the central part of the specimen (which is the reference case, Bui *et al.* 2014 [7]).

⇨ E_h_ = K∙E_1/3_, where K is the correction factor to correct modulus measured on specimen height.

For “old” specimens: E_h_^old^ = K∙E_1/3_^old^. For “new” specimens: E_h_^new^ = K∙E_1/3_^new^.

⇨ E_1/3_^new^/E_1/3_^old^ = E_h_^new^/E_h_^old^

Therefore, the results obtained by this method can be used to investigate the ageing phenomenon. Table 2 gives the results of this test. The modulus is calculated for stress levels between 0 and 20% of the maximum stress which represent the elastic part of RE material [7]. There is no important difference in moduli between the vertical and the horizontal directions of the wall (E ≈ 95 MPa). The strength of the specimens tested in the direction parallel to layers is slightly less than that of the specimens tested in the vertical direction (8%). No major difference in two directions’ moduli shows that the isotropic hypothesis which was assumed in the FEM (for small strains) is acceptable. This remark was noted in previous studies [9,10,13].

A good correlation of the moduli obtained by the dynamic and static methods can be observed—which are around 100 MPa—confirming the relevance of the results obtained.

**Table 2:** Results of the compressive strength and the elastic modulus obtained from the unconfined compression test.

Test direction	Density	Moisture content	Ultimate stress (MPa)	Ultimate strain	E (MPa)
Perpendicular to layers	1.82	1.4%	0.89 ± 0.10	0.013 ± 0.001	98 ± 6
Parallel to layers	1.82	1.3%	0.82 ± 0.08	0.012 ± 0.001	93 ± 5

## 4. Characterizing the “New” Rammed Earth

### 4.1. Manufacture of Specimens and Compression Tests

The soil of the specimen cut from the walls was recycled and reused for the manufacturing of the “new” specimens. In order to test the specimens in two directions (perpendicular and parallel to the layers), two types of specimens were manufactured:

- Two specimens (0.4 × 0.4 × 0.7) m^3^
- Two specimens (0.4 × 0.4 × 0.2) m^3^

**Figure 9:**
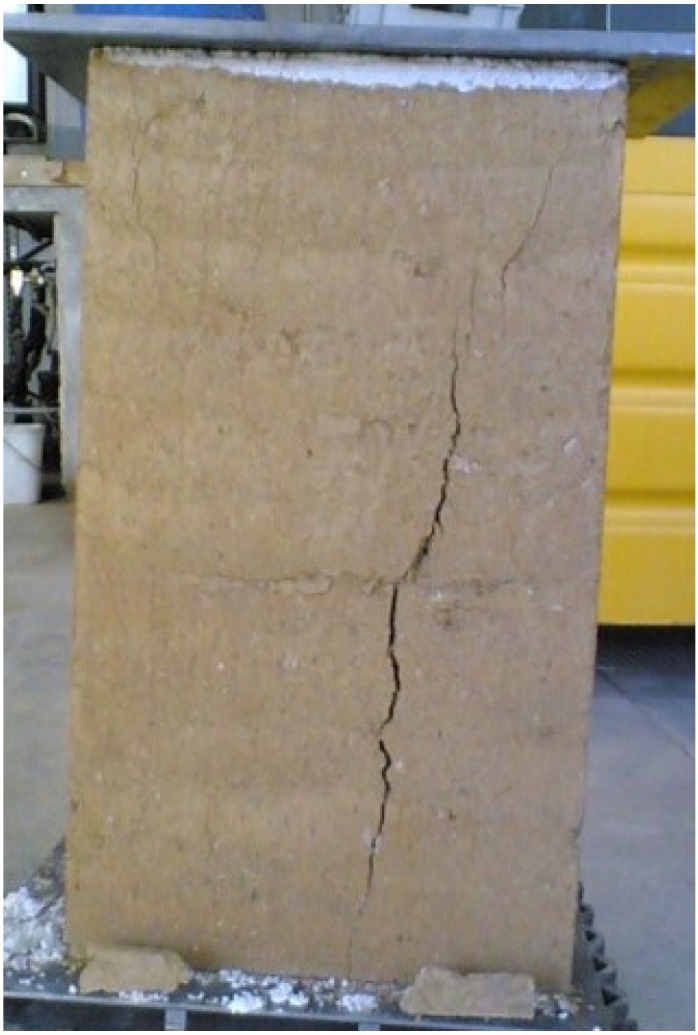
A specimen (40 × 40 × 70) cm^3^ after the compression test.

Discussions about the representativeness of specimens manufactured in laboratory were presented in the literature [1,9]. Indeed, to ensure a faithful representation of the *in-situ* wall material, the manufacturing mode and material used for laboratory specimens should be as identical as possible to those used *in situ*. The manufacturing water content and the compaction energy in the laboratory were chosen similar to that on site. The manufacturing water content was 10%. The specimens were rammed by an artisan of rammed earth with his pneumatic rammer. The dimensions of specimens tested in the direction perpendicular to the layers were 40 cm × 40 cm × 70 cm, with nine layers (Figure 9).

**Figure 10:**
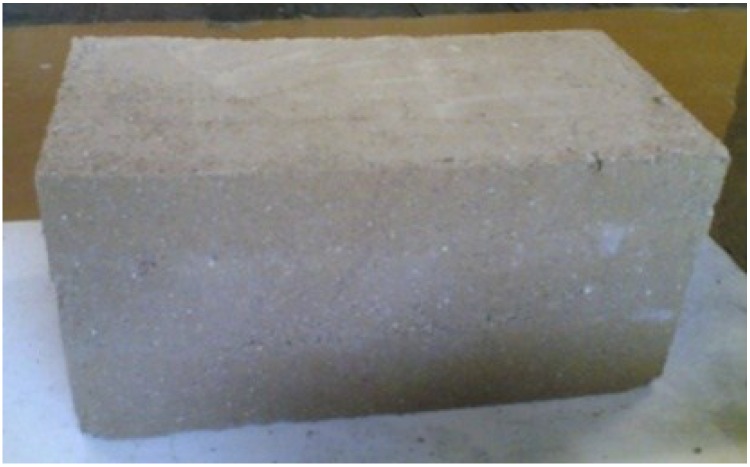
A specimen (20 × 20 × 40) cm^3^ cut from a specimen (40 × 40 × 20) cm^3^.

The specimens to be tested in the parallel direction were composed of three layers; their dimensions were 40 cm × 40 cm and 20 cm high. Special attention was given during compaction of the last layer to obtain a surface that was as flat as possible. To achieve a slenderness ratio of 2, the specimens were then cut with a table saw. Two specimens (40 × 40 × 20) cm^3^ provided four specimens (20 × 20 × 40) cm^3^ for testing in the parallel direction (Figure 10). For specimens tested in the direction parallel to the layers, a surfacing was not necessary, because the two surfaces that were in contact with the formwork were sufficiently flat.

### 4.2. Results

The results are presented in the Table 3. Once again, there is no significant difference between the elastic moduli of the vertical and horizontal directions of the wall (E ≈ 270 MPa). The compressive strength of the specimens tested in the direction parallel to layers is slightly less than that of the specimens tested in the perpendicular direction (8%).

**Table 3:** Results of compression tests on the new specimens.

Test direction	Density	Moisture content	Ultimate stress (MPa)	Ultimate strain	E (MPa)
Perpendicular to layers	1.91	1.8%	1.35 ± 0.1	0.008 ± 0.001	263 ± 12
Parallel to layers	1.91	1.7%	1.18 ± 0.1	0.007 ± 0.001	287 ± 8

## 5. Discussion and the Creep Coefficient

Except for the case of exterior walls, the *in-situ* walls in this study are not directly representative of current RE houses. Indeed, they have two outer surfaces, whereas in a house, the interior atmosphere (without rain) is different from the external one subjected to weathering.

The strategy which studies the initial state by manufacturing the new specimens from the recycled soil is questionable because the new ones are similar but not the same as the initial state ones. On one hand, are there possible changes in the soil’s characteristics after 22 years on site due to cycles of adsorption-desorption and freeze-thaw? On the other hand, the new specimens have a slightly higher dry density than that of the old walls. There are two possible reasons for this: firstly, compaction energy which depends on the artisan experience is higher in the case of the new specimens. Secondly, there is a possible change in the porosity of the walls due to the adsorption-desorption and freeze-thaw cycles [25,26]. If the second reason is confirmed, it will be an interesting element in investigations on the ageing effects of rammed earth walls.

Due to the difference of the dry density (about 5%) between the old walls and the new specimens, there is a difference in the corresponding compressive strength (about 50%). This result is not surprising and was observed in the literature [27]. The interesting remark is the difference in the Young moduli: the new specimens had moduli which were 2.7 times greater than the ones of the old walls (instead of 1.5 for the compressive strength). We analyse the difference in stiffness:

- damage of the sample during the “old” specimens’ taking: however, the *in-situ* dynamic tests give the similar results of modulus. So, it is not this possibility.
- Problem of representativeness of the “new” specimens: there are also possible differences in the compaction energy, the manufacturing water content between the “old” walls and the “new” specimens. Although the walls and the new specimens were all manufactured by the rammed earth professionals, a difference is not evitable. However, in our opinion, this difference cannot be the only factor which can cause a significant difference in the dry density.
- Propagation of micro-cracks under weathering loadings: there are changes at the micro-structure of the material: micro-pores increase which lead to a decrease of dry density.

So, the second and third reasons are the probable factors. The third is the ageing phenomenon which has a link to the creep phenomenon. Indeed, for concrete, the creep occurs at all stress levels and, within the service stress range, is linearly dependent on the stress if the pore water content is constant [24]. For rammed earth, the increase of strain under a constant stress was noted in Lombillo *et al.* study [28] and compared to the phenomenon of consolidation of normally consolidated soil.

The creep depends on the ambient humidity, composition of the RE material, age of material, duration and intensity of the loading. In the case of the studied walls, the creep due to the loading was negligible because the stress levels were low but the creep due to the weathering could take place.

For concrete, following Eurocode 2 [24], the effective modulus *E_eff_* is related to the initial modulus *E_t_*_0_ (at 28 days) by the formula:

*E_eff_* = *E_t_*_0_/[1 + φ] (4)

where φ is the creep coefficient,

φ = *E_t_*_0_*/E_eff_* – 1 (5)

If the modulus of the walls and the new specimens are used respectively for *E_eff_* and *E_t_*_0_, the corresponding creep coefficient of the walls is 1.7. This information is interesting because, to our knowledge, this is the first time a value of RE creep coefficient is presented.

In the last decade, to explain the basic creep of concrete, physical mechanisms taking action at the scale of the hydrates were proposed; they are based on the microprestress-solidification theory, the viscoplastic behaviour of the hydrates (principally the C–S–H which is the principal component of the cement), and the rearrangement of nanoscale particles (C–S–H level) following the free-volume dynamics theory of granular physics (a synthesis can be found in Rossi *et al.* [29]). However, in the case of RE, these theories cannot explain its creep because there are not C–S–H particles. A recent proposal by Rossi *et al.* [29] showed that even other physical mechanisms can exist; the main physical origins of the basic creep are related to the microcracking propagation under load. In the case of RE, when a wall is under a loading (self-weight, wind, temperature, freeze-thaw), it cracks. The microcracks generate a severe hygric imbalance within the material. Indeed, creation of microcracks provokes the appearance of vacuums effect (gradients of pressure) and local hygric shocks (gradients of concentration in water molecule). That is why there is a propagation of the initial microcracks. The microcrack propagation is suggested to be the main factor for the decrease of the Young modulus of RE material. The decrease of the Young modulus due to the increase of the porosity had been reported in the Wang and Li study (2007) for concrete material [30].

## 6. Conclusions and Outlook

In this study, the ageing effects on 22 years old RE walls were studied. Mechanical characteristics of these “old” walls were determined by *in-situ* dynamic measurements and by laboratory compression tests. Then, soil was reused to manufacture the new specimens with the same way as the old walls.

There are several factors which influence the creep phenomenon: Material and ambiance: the composition of the material; the rate of hardening of the material; the dimensions of the element; ambient humidity; ambient temperature; Loading: age of the material at loading; the duration of the loading and the stress level. In the case of the studied walls, the creep due to the loading was negligible because the stress levels were low but the creep due to the weathering was observed.

The strategy which studies the initial state by manufacturing the new specimens from the recycled soil is questionable. However, it is always interesting to have direct information from real walls exposed to natural conditions. For the walls studied in this paper, a creep coefficient was obtained. However, this is a first exploratory study on the ageing and creep of RE walls, so the results should be confirmed by other studies in the future. Two other approaches are planned the next time: first, other new specimens will be manufactured to have the same dry density as the old walls; second, acceleration tests [29] which are currently used for creep studies of concrete will be applied.
